# Distal purse-string suture technique for TaTME

**DOI:** 10.1007/s10151-018-1917-8

**Published:** 2018-12-22

**Authors:** R. Wu, R. Benedict, A. Caycedo-Marulanda

**Affiliations:** 1 Health Sciences North, 65 Larch St. Suite 308, Sudbury, ON P3E 1B8 Canada; 2 Northern Ontario School of Medicine, Sudbury, ON Canada; 3 Colorectal Surgery North, Sudbury, ON Canada

Transanal total mesorectal excision (taTME) is a novel technique that offers the possibility of overcoming the challenges of accessing the deep pelvis, when performing radical rectal surgery. It allows the surgeon to perpendicularly divide the rectum and precisely select the distal margin. Evidence has demonstrated that extensive training and proctoring are recommended to reach proficiency [1].

TaTME has a number of key steps. This operation continues to accrue evidence in favor of good outcomes; however, it has proven to be a formidable technical challenge for surgeons to master this approach. In the existent literature, there are reports regarding the options to create an anastomosis during taTME where bowel restoration is appropriate [2].

There are no publications on the technical aspects of creating the distal purse-string, which plays a critical role when it comes to ensure the integrity of the anastomosis among other factors.

In our institution, we have accumulated significant experience on this procedure [3]. In doing so, we have standardized our taTME technique, including the distal purse-string suture.

Important elements of the purse-string suture include: (1) loading the needle at a 30° angle on the needle driver; (2) full thickness bites of the rectal wall, no more than 5 mm apart; (3) preventing redundancy in the suture before placing the next stitch; (4) avoiding holding the needle with both hands simultaneously to minimize torque on the rectal wall; (5) continuous awareness of the surrounding structures to avoid incorporating them into the anastomosis; (6) confirming the quality of the suture purchase via the laparoscopic view where possible; and (7) placement of the last stitch in reverse to bring both ends of the suture to the same side. We firmly believe that the adherence to these steps has helped ensure good patient outcomes.

We have found by experience that this technique is facilitated using an end-curved needle holder such as the Jarit needle holder manufactured by Integra Life Sciences.

The following video demonstrates our technique with these elements, for the placement of the distal purse-string suture in preparation for restoration of bowel continuity.

## Electronic supplementary material

Below is the link to the electronic supplementary material.

Supplementary material 1 (MP4 418724 KB)
